# Hyaluronic acid-CD44 interactions promote BMP4/7-dependent Id1/3 expression in melanoma cells

**DOI:** 10.1038/s41598-018-33337-7

**Published:** 2018-10-08

**Authors:** Ruo-Lin Wu, Georg Sedlmeier, Lenka Kyjacova, Anja Schmaus, Julia Philipp, Wilko Thiele, Boyan K. Garvalov, Jonathan P. Sleeman

**Affiliations:** 1 European Center for Angioscience (ECAS), Medical Faculty of Mannheim, Heidelberg University, 68167 Mannheim, Germany; 2 KIT Campus Nord, Institute for Toxicology and Genetics, 76344 Karlsruhe, Germany; 3 Present Address: Department of Hepatopancreatobiliary Surgery and Organ Transplantation Center, First Affiliated Hospital of Anhui Medical University, Hefei, 230022 Anhui China

**Keywords:** Melanoma, Extracellular signalling molecules, Glycobiology

## Abstract

BMP4/7-dependent expression of inhibitor of differentiation/DNA binding (Id) proteins 1 and 3 has been implicated in tumor progression and poor prognosis of malignant melanoma patients. Hyaluronic acid (HA), a pericellular matrix component, supports BMP7 signalling in murine chondrocytes through its receptor CD44. However, its role in regulating BMP signalling in melanoma is not clear. In this study we found that depletion of endogenously-produced HA by hyaluronidase treatment or by inhibition of HA synthesis by 4-methylumbelliferone (4-MU) resulted in reduced BMP4/7-dependent Id1/3 protein expression in mouse melanoma B16-F10 and Ret cells. Conversely, exogenous HA treatment increased BMP4/7-dependent Id1/3 protein expression. Knockdown of CD44 reduced BMP4/7-dependent Id1/3 protein expression, and attenuated the ability of exogenous HA to stimulate Id1 and Id3 expression in response to BMP. Co-IP experiments demonstrated that CD44 can physically associate with the BMP type II receptor (BMPR) ACVR2B. Importantly, we found that coordinate expression of Id1 or Id3 with HA synthases HAS2, HAS3, and CD44 is associated with reduced overall survival of cutaneous melanoma patients. Our results suggest that HA-CD44 interactions with BMPR promote BMP4/7-dependent Id1/3 protein expression in melanoma, contributing to reduced survival in melanoma patients.

## Introduction

Bone morphogenetic proteins (BMPs) belong to the transforming growth factor β (TGF-β) superfamily. They signal through specific type I and II serine/threonine receptors, which in turn activate Smad-1/5/8 transcription factors. After this activation, Smad proteins form heteromeric complexes with the common-mediator Smad4 and translocate into the nucleus, where they regulate gene transcription^1^. The inhibitor of differentiation/DNA binding (Id) genes Id1 and Id3 are well-characterized target genes of the BMP signalling pathway^1^. The four members of the Id family (Id1-4) are helix-loop-helix proteins that lack a DNA-binding domain, and act as dominant-negative antagonists of basic helix-loop-helix transcription factors by forming inactive heterodimers^2^. Through this activity Id proteins regulate a variety of cellular processes including proliferation, migration, invasion, angiogenesis, maintenance of stem cell properties, and cell differentiation^3,4^. Moreover, Id1 and Id3 endow tumor cells with stemness properties, thereby contributing to cancer initiation *in vivo*^4–8^. Id1 and Id3 are closely related, have overlapping biological functions, and can partially compensate for each other^4,9^.

Increased expression of BMPs such as BMP4 and BMP7 correlates with poor prognosis in various cancers^10^. As direct targets of BMP signaling^11–13^, Id genes are likely to be key mediators of the prognostic effects of BMPs. Notably, Id genes such as Id1 and Id3 also correlate with poor prognosis in many cancer types^4^.

In the context of malignant melanoma, BMP7 expression correlates with poor prognosis^14^. Increased levels of BMP4 and BMP7 also promote cell migration and invasion of human melanoma cells *in vitro*^15^. Consistently, increased Id1 expression has been correlated with tumor progression and poor prognosis in malignant melanoma patients^16^. Id3 expression has also been shown to promote tumorigenesis in melanoma^17^. Thus the BMP/Id axis contributes to the malignancy of melanoma.

The ability of BMPs to activate their receptors is regulated by a number of extracellular matrix (ECM) components^1^. For example, hyaluronan (HA) is a linear high molecular weight polysaccharide in the ECM that is composed of repeating disaccharides of β-1-3, β-1-4-linked glucuronic acid and N-acetylglucosamine, and which has been implicated in the regulation of BMP signalling in chondrocytes^18^. Three transmembrane synthases (HAS1, HAS2, HAS3) are responsible for HA synthesis, and extrude HA into the pericellular matrix. The biological functions of HA are mainly mediated by cell surface receptors, in particular CD44^19^. CD44 acts as a co-receptor for a number of cell surface growth factors and cytokine receptors, and its ability to act as a co-receptor can be regulated by HA^20^. In chondrocytes, CD44 interacts intracellularly with Smad1, and promotes Smad1 and Smad4 phosphorylation and nuclear translocation in response to BMP7 in HA-dependent manner^18,21^.

High levels of CD44 correlate with cancer progression and poor survival in melanoma patients^22^. Melanoma cells produce large amounts of HA during early tumorigenesis, whereas in later stages, HA is largely produced by activated tumor-associated stromal fibroblasts^23–25^. Thus melanoma cells are exposed to both self-produced HA and stroma-derived HA during tumor progression. In melanoma, HA has been implicated in promoting cell proliferation^25,26^, adhesion^27^, motility^28,29^, invasion, and metastasis^30^. In the B16-F10 murine melanoma model, inhibition of HA synthesis using 4-MU reduced cell adhesion and locomotion^31^.

Given that the CD44/HA axis promotes BMP signalling in chondrocytes, and the correlation between these factors and poor prognosis in melanoma, we hypothesized that HA might promote BMP4/7-dependent Id1/3 protein expression in melanoma cells in CD44-dependent manner. In this study, we investigated this hypothesis, and found that the CD44/HA axis fosters BMP4/7-dependent Id1/3 protein expression in melanoma cells through interactions between CD44 and BMPR. Consistently, coordinate expression of Id1 or Id3 with HAS2, HAS3 or CD44 significantly correlated with reduced survival of cutaneous melanoma patients. We therefore conclude that the CD44/HA axis can potentiate the ability of BMP4/7 to activate Id1 and Id3 expression in melanoma cells, thereby contributing to poor prognosis.

## Results

### BMP4/7 stimulation induces Id1/3 protein expression in mouse melanoma cells

We have previously shown that BMP4 and BMP7 both induce Id1/3 protein expression in B16-F10 and Ret cells, with peak induction of Id1/3 protein occurring 6 hours after the stimulation^32^. In further experiments we found that the expression of Id1/3 proteins was increased by BMP4/7 stimulation in a dose-dependent manner (Supplementary Fig. S1A). We also determined the effect of BMP4 and BMP7 on proliferation of the melanoma cells. As shown in Supplementary Fig. S1B, low concentrations of BMP4/7 had no significant effect on cellular proliferation, whereas high concentrations showed an inhibitory effect. These data suggest that a low concentration of BMP4/7 (10 ng/ml) is sufficient to induce the expression of Id1/3 proteins without having a significant inhibitory effect on cell proliferation. This concentration was therefore used for subsequent experiments.

### Hyaluronidase digestion reduces BMP-dependent Id1/3 protein expression

**Figure 1:**
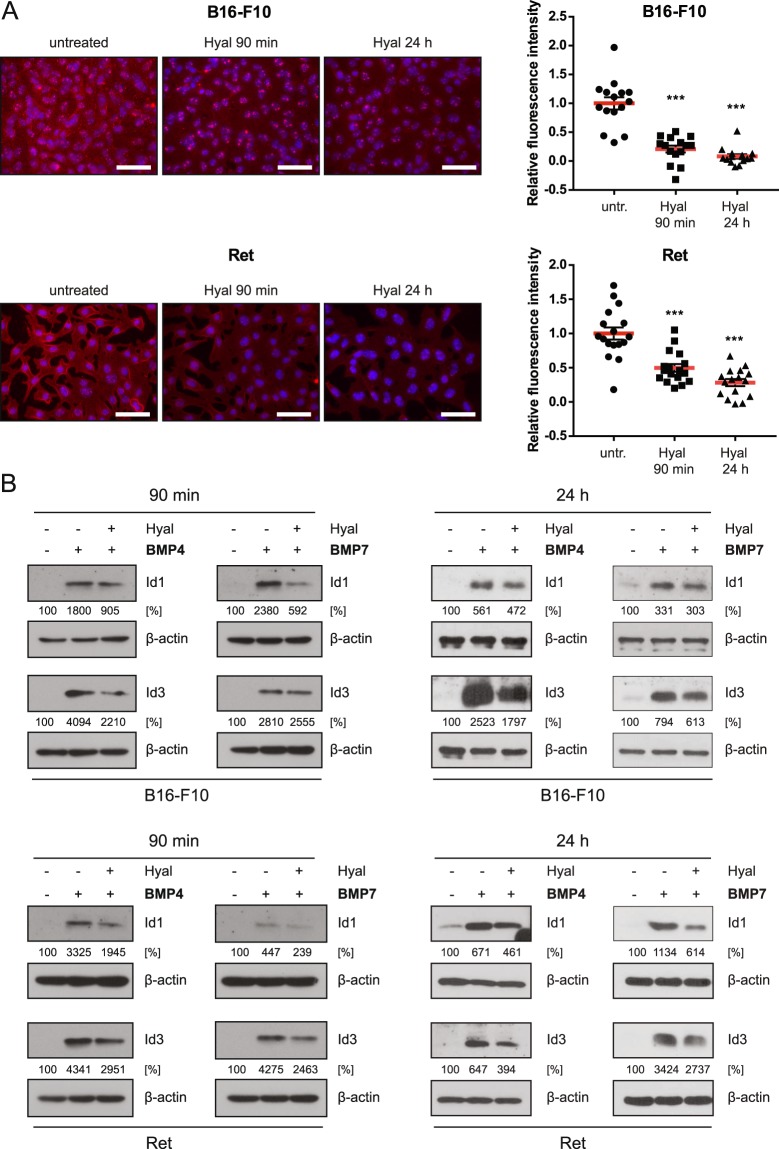
Depletion of HA in the pericellular matrix reduces BMP-dependent Id1/3 protein expression in murine melanoma cells. (**A**) B16-F10 and Ret cells were treated with 100 rTU bovine testis hyaluronidase (Hyal) for 90 min or 24 hours. Hyaluronic acid was detected using HABP immunofluorescence staining. HABP staining intensity from at least 15 images was quantified using ImageJ in a blinded fashion. Scale bar 50 μm. Student’s t-test: ***p < 0.001. (**B**) B16-F10 and Ret cells were treated or not treated with 100 rTU hyaluronidase for 90 min or 24 hours, medium was changed, and then stimulated with 10 ng/ml recombinant BMP4/7 for another 6 hours. Cell lysates were analyzed for Id1/3 protein expression by Western blot. β-actin was used as a loading control. Densitometry analysis was performed using ImageJ software. Numbers indicate integrated density relative to untreated controls.

The presence of HA in the pericellular matrix has been shown to foster the BMP7 signaling pathway in chondrocytes^18,21,33^. We therefore investigated whether HA in the pericellular matrix might play a similar role in regulating BMP-induced Id1/Id3 expression in B16-F10 and Ret melanoma cells. To this end, HA in the pericellular matrix was depleted by hyaluronidase (Hyal) treatment. For both cell lines, significantly reduced levels of pericellular HA were detected by HABP staining after treatment with exogenous hyaluronidase (Fig. 1A). In subsequent experiments, cells were treated with and without hyaluronidase prior to the stimulation with BMP4/7. Hyaluronidase pre-treatment reduced Id1/3 protein expression in response to BMP4 and BMP7 in both melanoma cell lines by around 50% (Fig. 1B). These results suggest that HA in the pericellular matrix is required for efficient Id1/3 protein expression in response to BMP stimulation.

### 4-methylumbelliferone (4-MU) treatment reduces BMP-dependent Id1/3 protein expression

**Figure 2:**
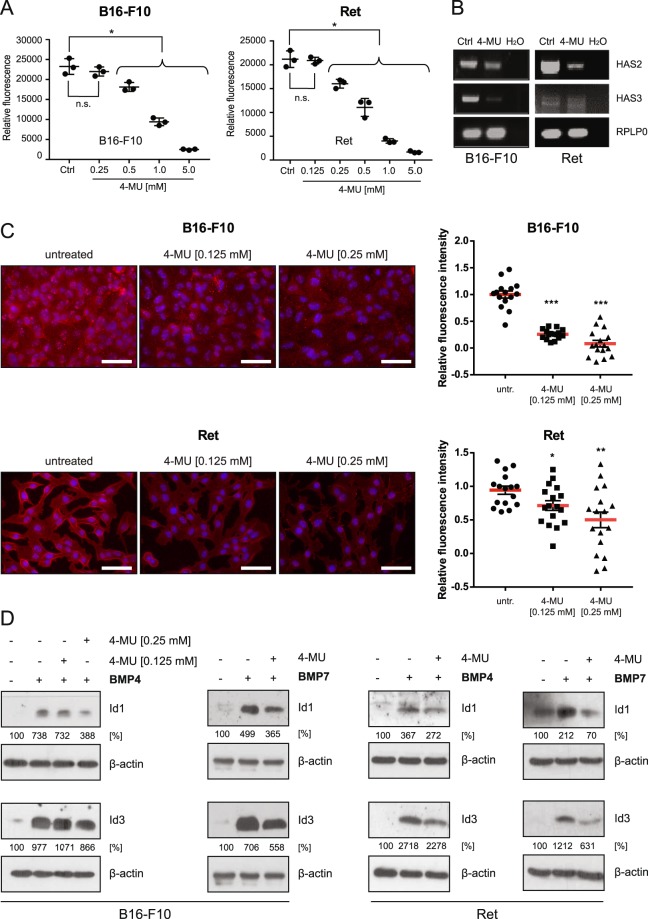
Inhibition of HA synthesis reduces BMP-dependent Id1/3 protein expression in murine melanoma cells. (**A**) B16-F10 and Ret cells were treated with the indicated concentrations of 4-methylumbelliferone (4-MU) for 48 hours. The number of cells was then quantified on the basis of DNA content using CyQUANT assay. The mean and SE of triplicate samples are shown. Data represents means ± SD, n = 3. Student’s t-test: *p < 0.05; n.s., non-significant. (**B**) B16-F10 and Ret cells were treated with 0.25 mM (B16-F10) and 0.125 mM (Ret) 4-MU for 48 hours. Total RNA was prepared, and RT-PCR was performed to analyze the gene expression of HAS2 and HAS3. For a negative control, water instead of cDNA was added to the PCR reaction (H_2_O). Rplp0 was used as an internal loading control. (**C**) B16-F10 and Ret cells were treated or not treated with the indicated concentrations of 4-MU for 48 hours. Hyaluronic acid was detected using HABP immunofluorescence staining. HABP staining intensity was quantified using ImageJ. At least 15 images were quantified in a blinded fashion. Scale bar 50 μm. Student’s t-test: *p < 0.05, **p < 0.01, ***p < 0.001. (**D**) B16-F10 and Ret cells were treated or not treated with 4-MU (0.25 mM, B16-F10, right; 0.125 mM, Ret) for 48 hours, then stimulated with 10 ng/ml recombinant BMP4/7 for another 6 hours. Cell lysates were then analyzed for Id1/3 protein expression by Western blot. β-actin was used as a loading control. Densitometric quantification is shown below the Western blots, representing the signal normalized to the loading control (β-actin), relative to the untreated controls.

To further substantiate the notion that HA promotes BMP4/7-induced Id1/Id3 expression in melanoma cells, we treated the cells with 4-MU, a 7-hydroxy-4-methylcoumarin that inhibits HA synthesis by depleting cellular UDP-glucuronic acid and downregulating HA synthase expression^34^. To exclude the possibility that 4-MU affects cell viability or proliferation in these experiments, we first determined whether 4-MU treatment has any direct effects on cell numbers. As shown in Fig. 2A, the number of cells decreased in a concentration-dependent manner when the concentrations of 4-MU exceeded 0.25 mM and 0.125 mM for B16-F10 and Ret cells, respectively. Importantly, expression of HA synthase (HAS) 2 and 3 was downregulated after the treatment of the cells with concentrations of 4-MU that did not reduce cell numbers (Fig. 2B), which corresponded to reduced amounts of HA in the pericellular matrix (Fig. 2C). Note that little or no expression of HAS1 was detected in the cells used in this study (Supplementary Fig. S2).

To investigate whether inhibition of endogenous HA synthesis influences BMP4/7-induced Id1/3 protein expression, cells were stimulated with BMP4/7 with or without pre-treatment with 4-MU. BMP4/7-induced Id1/3 protein expression was reduced by 4-MU treatment by around 50% (Fig. 2D). These findings suggest that inhibition of endogenous HA synthesis reduces Id1/3 protein expression in response to BMP stimulation.

### Exogenous HA augments BMP-dependent Id1/3 protein expression

**Figure 3:**
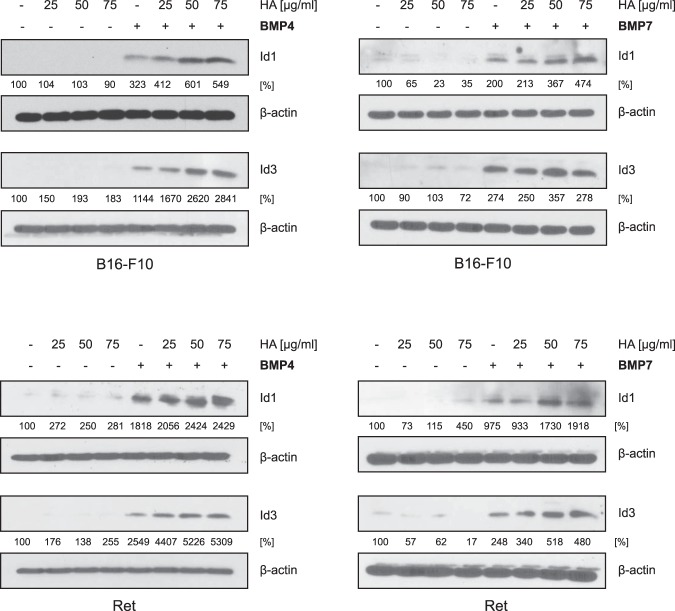
Exogenous HA promotes BMP-dependent Id1/3 protein expression in murine melanoma cells. B16-F10 and Ret cells were treated with the indicated concentrations of HA for 24 hours, then stimulated with 10 ng/ml recombinant BMP4/7 for another 6 hours. Cell lysates were then analyzed for Id1 and Id3 protein expression by Western blot. β-actin was used as loading control. Densitometric quantification is shown below the Western blots, representing the signal normalized to the loading control (β-actin), relative to the untreated controls.

HA is produced not only by melanoma cells, but also by other cells in their environment such as fibroblasts. To determine whether HA from such exogenous sources can also potentiate BMP-dependent Id1/3 protein expression, B16-F10 cells and Ret cells were stimulated with BMP4/7 with or without pre-incubation with various concentrations of HA. Pre-incubation with HA modestly augmented BMP4/7-induced Id1/3 protein expression when compared to non-treated control cells (Fig. 3). These data again indicate that HA can promote BMP4/7-dependent Id1/3 protein expression.

### Knockdown of CD44 reduces BMP-dependent Id1/3 protein expression

**Figure 4:**
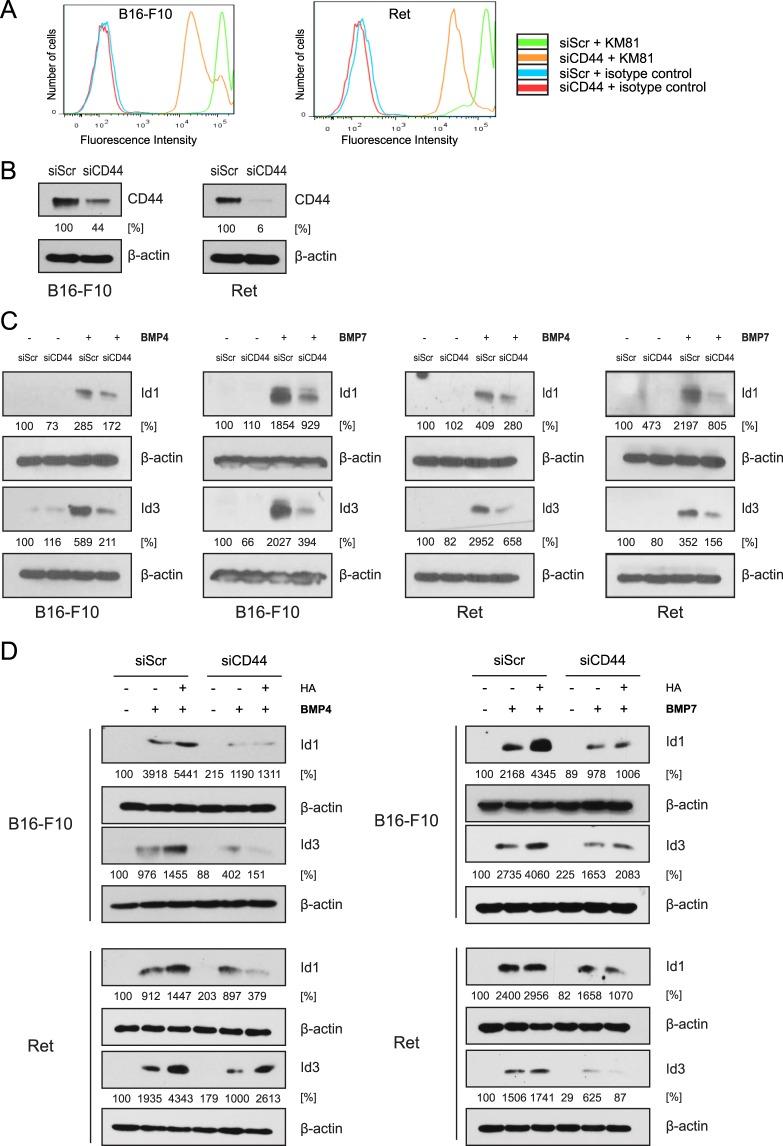
Knockdown of CD44 reduces BMP-dependent Id1/3 protein expression in murine melanoma cells. (**A**) B16-F10 and Ret cells were transfected with CD44 siRNA (siCD44) or scrambled control siRNA (siScr) for 24 hours (CD44 siRNA group, yellow histogram; scrambled siRNA group, blue histogram). Cells were incubated with the anti-CD44 mAb KM81 and surface expression of CD44 was measured by FACS analysis. Isotype-matched control IgG antibody (red and blue histogram) served as a control. (**B**) Western blot detection of CD44 after the siRNA-mediated CD44 knockdown (siCD44). Scrambled siRNA (siScr) was used as a control. β-actin was used as a loading control. (**C**) B16-F10 and Ret cells were transfected with CD44 siRNA (siCD44) or scrambled siRNA (siScr) for 24 hours, then stimulated with 10 ng/ml recombinant BMP4/7 for another 6 hours. Cell lysates were then analyzed for Id1/3 protein expression by Western blot. β-actin was used as a loading control. (**D**) B16-F10 and Ret cells were transfected with CD44 siRNA or scrambled siRNA. 24 hours after transfection, cells were incubated with HA for 24 hours, then stimulated with 10 ng/ml recombinant BMP4/7 for another 6 hours. Cell lysates were then analyzed for Id1/3 protein expression by Western blot. β-actin was used as a loading control. Densitometric quantification is shown below the Western blots, representing the signal normalized to the loading control (β-actin), relative to the untreated controls.

Next we determined whether CD44, the principal cell surface receptor for HA, plays a role in the ability of HA to stimulate BMP-induced Id1 and Id3 expression in B16-F10 and Ret melanoma cells. Using FACS analysis, surface expression of CD44 was detected at high levels in both melanoma cell lines (Fig. 4A). Western blot analysis showed that virtually all CD44 expressed in the cells is the standard 85 kDa isoform (Supplementary Fig. S3). CD44 expression was knocked down using siRNA (Fig. 4A,B). In comparison to scrambled siRNA controls, CD44 knockdown was found to reduce Id1/3 expression in response to BMP4 and BMP7 (Fig. 4C). This finding suggests that the interaction between HA and its surface receptor CD44 contributes to BMP-dependent Id1/3 expression. Consistent with this notion, knockdown of CD44 abrogated the ability of exogenously added HA to stimulate Id1/3 expression in response to BMP, with the exception of Id3 expression in Ret cells following BMP4 treatment (Fig. 4D).

### CD44 and the BMP receptor ACVR2B physically interact

**Figure 5:**
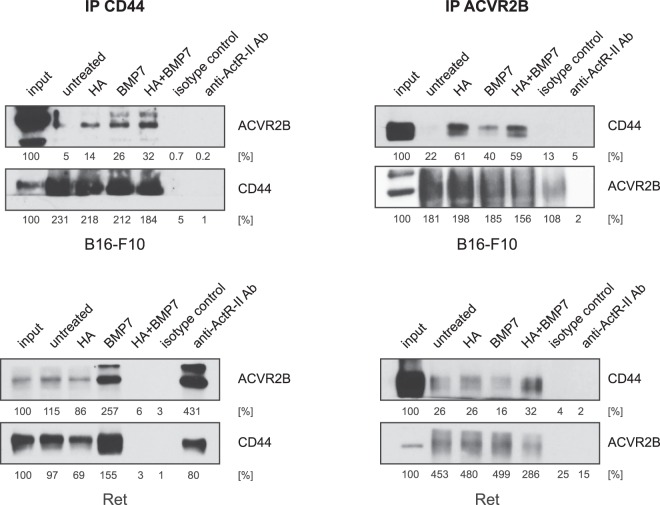
CD44 and ACVR2B can physically interact in melanoma cells. B16-F10 and Ret cells were pre-incubated with HA (50 µg/ml) for 48 hours, and then stimulated with or without BMP7 (10 ng/ml) for another 6 hours. Cell lysates were then analyzed for CD44 and ACVR2B protein expression by co-immunoprecipitation assays. In each case, the samples in the two left panels were immunoprecipitated with CD44 antibodies, and the samples in the right panels were immunoprecipitated with ACVR2B antibodies. The Western blots were probed with either CD44 or ACVR2B antibodies as indicated. Isotype control antibodies were used for negative control immunoprecipitations. Densitometric quantification is shown below the Western blots, representing the signal relative to the input lanes.

Next we used qRT-PCR to determine which BMP receptors are expressed on Ret and B16-F10 cells. As shown in Supplementary Fig. S4, both cell lines express ALK3 and ACVR2B as the major type I and type II receptors, respectively. To investigate whether CD44 can act as a co-receptor for BMP in melanoma cells, co-IP of CD44 and ACVR2B was performed. As shown in the left panels of Fig. 5, immunoprecipitation of CD44 resulted in the co-IP of ACVR2B in B16-F10 and Ret cells. In the right panels of Fig. 5, immunoprecipitation of ACVR2B resulted in the co-IP of CD44 in both cell lines. In B16-F10 cells, we observed no co-IP between ACVR2B and CD44 in the absence of HA or ligand, while HA and BMP7 both stimulated complex formation between ACVR2B and CD44. In Ret cells, a weak association between ACVR2B and CD44 was observed in the absence or presence of HA or BMP7 individually, which was strongly augmented in the presence of HA and BMP7 together. These data suggest that HA can promote BMP4/7-dependent Id1 and Id3 protein expression through promoting the interaction between CD44 and ACVR2B, with the exact degree of interaction being dependent on the cellular context and the presence or absence of ligands.

### Coordinate expression of HAS and Id genes in human melanoma correlates with reduced survival

**Figure 6:**
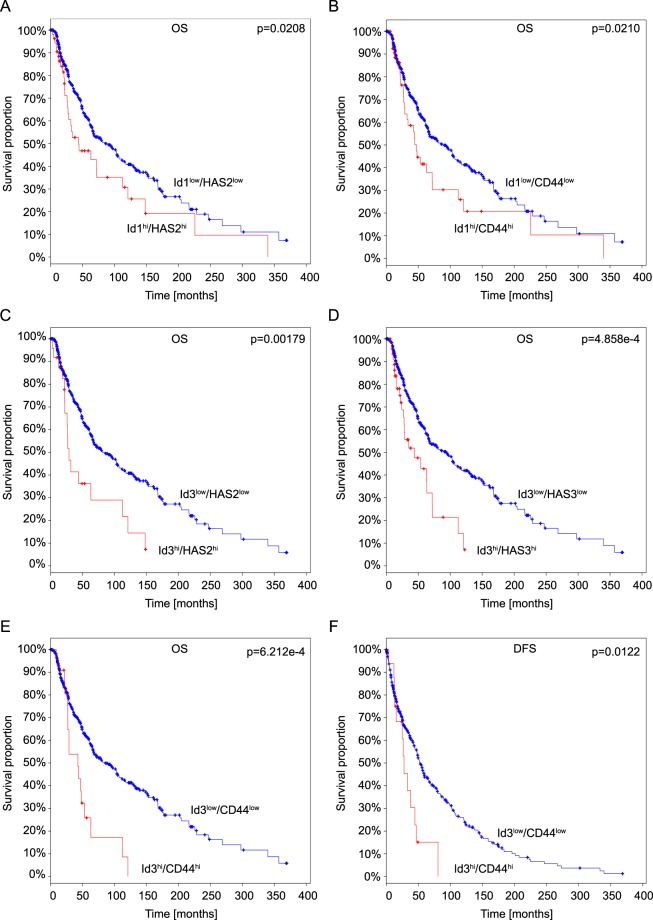
Analysis of the TCGA Cutaneous Melanoma datasets (SKCM, provisional) shows that increased expression of Id1 or Id3 with HAS or CD44 genes is associated with poor prognosis in cutaneous melanoma patients. Kaplan-Meier analysis of overall survival (**A**–**E**) and disease-free survival (**F**) of 479 patients with cutaneous melanoma from the TCGA cohort (TCGA, provisional) whose tumors express different levels of Id1 or Id3 with HAS or CD44 mRNA. A z-score threshold of 2.0 was used to discriminate between tumors with low and high (hi) expression of the indicated genes. Survival curves were plotted using the cBio portal^35,36^.

Our data using cultured cell lines suggest that increased levels of HA could potentially contribute to poor prognosis by stimulating Id expression in a CD44/HA-dependent manner. To determine whether these findings might have relevance in the context of human melanoma, we analysed mRNA expression data in the cancer genome atlas (TCGA) database^35,36^ derived from cutaneous melanomas, to assess whether coordinate expression of Id1 and/or Id3 with HAS genes or CD44 correlates with reduced survival. Coordinate expression of Id1 and HAS2 as well as Id1 and CD44 both correlated with reduced overall survival (Fig. 6). Similarly coordinate expression of Id3 with HAS2, HAS3 or CD44 was also associated with reduced overall survival. Furthermore, coordinate expression of Id3 and CD44 correlated with significantly reduced disease-free survival (Fig. 6). These data are consistent with the notion that the CD44/HA axis and Id1/Id3 expression have protumorigenic roles in human melanoma, resulting in reduced survival.

## Discussion

Here we report that HA promotes BMP4/7-dependent Id1/3 protein expression in melanoma cells. Specifically we show that degradation of HA in the pericellular matrix or inhibition of endogenous HA synthesis reduces BMP4/7- dependent Id1/3 protein expression, while exogenous HA has the opposite effect. CD44, the main cell surface receptor for HA, physically interacts with the BMP receptor ACVR2B, and is required for the ability of HA to promote BMP-induced Id1/Id3 expression. Coordinate expression of Id1 or Id3 or CD44 with HAS2 or HAS3 or CD44 was found to be associated with reduced survival of human melanoma patients. Together these findings identify a novel role for HA in the regulation of BMP signaling pathway in melanoma that has prognostic significance.

CD44 is as a multi-domain signaling platform that integrates extracellular matrix cues with growth factor and cytokines signals^20^. CD44 exerts a co-receptor function for a variety of receptor tyrosine kinases including Met, VEGFR-2, PDGFR, and EGFR through physically interacting with these cell surface proteins in *cis*, thereby promoting their ligand-dependent activation. Although alternatively spliced CD44 variants often act as co-receptors, the CD44s isoform interacts with ErbB2 and ErB3 in Schwann cells, and thereby acts as a co-receptor for neuregulin^37^, and also acts to stimulate TGF-βRI activation^38^. Here we show that CD44s also physically interacts with the BMP receptor ACVR2B and provide evidence that it acts as a co-receptor for BMPs, as knockdown of CD44 reduced BMP-dependent Id1/Id3 expression. As the cytoplasmic portion of CD44 interacts with Smad1 in chondrocytes, and promotes phosphorylation of Smad1 and Smad4 and their nuclear translocation^18,21^, it is likely that a similar mechanism underlies the BMPR co-receptor function for CD44 in melanoma cells.

We show here that HA promotes BMP4 and BMP7 signaling, as evidenced by increased Id1 and Id3 expression, in a CD44 dependent manner. A dependence on CD44 for BMP4-induced Id3 expression in Ret cells was not observed, suggesting that the activity of other signaling pathways may cross-regulate Id3 expression in these cells. In chondrocytes, HA has been shown to potentiate BMP7-induced phosphorylation and nuclear localization of Smad1 and Smad4^18,21^. HA can also cross-regulate the co-receptor function of CD44 in other context. For example, HA potentiates the ability of CD44s to activate TGF-βRI^38^. Conversely, HA inhibits ligand-induced PDGF β-receptor activation by fostering the formation of a physical complex between CD44s and PDGF β -receptor, in which CD44s probably recruits a negatively acting phosphatase to the PDGFβ-receptor^39^. CD44-dependent CXCL12-induced activation of CXCR4 is positively regulated by high molecular weight HA, but is negatively regulated by small HA oligosaccharides^40^. Similarly, high molecular weight HA fosters ErbB2 phosphorylation in a CD44-dependent manner, while small HA oligosaccharides inhibit ErbB2 phosphorylation^41^. Here we show that high molecular weight HA stimulates CD44-dependent BMP-induced expression of Id1 and Id3.

Id1 and Id3 proteins are overexpressed in a variety of human cancers, and play critical roles in cancer development and progression^4^. The tumor-promoting properties of these transcriptional regulators, and the ability of the HA/CD44 axis to promote their expression in response to BMP stimulation, likely underlies the association between coordinate expression of Id1/Id3 and HAS2/HAS3/CD44, and reduced survival of melanoma patients. Increased BMP-induced Id1/Id3 expression in response to HA could act at a number of levels to promote melanoma formation and progression. Id1/Id3 expression promotes stemness properties and tumor initiation^4^. Both BMP4 and BMP7 are present in the skin^42,43^, and the skin is a HA-rich organ^44^. Thus the augmentation of BMP-induced Id1 and Id3 expression by HA may be important for the genesis of melanoma once BMP-mediated growth inhibition has been overcome^45^. In addition, if disseminating melanoma cells settle in an HA-rich microenvironment with only low concentrations of BMPs, then melanoma metastasis formation may be fostered by HA-stimulated BMP-induced Id1/Id3 expression due to the tumor-initiating properties endowed on cancer cells by Id1 and Id3.

In this study, we have focused on melanoma. Nevertheless the mechanism of HA-stimulated BMP-induced Id1/Id3 expression may be relevant for other cancers, given that Id expression correlates with poor prognosis for a broad range of tumor types^4^. We have also focused on only one type II BMP receptor, ACVR2B. Future work will determine whether the activity of other BMP receptors is also fostered by the HA/CD44 axis.

Id1 and Id3 proteins are considered to have overlapping biological functions, and can compensate for each other. Therefore, inhibition of these Id proteins singly might not be sufficient to achieve anticancer effects. Our study reports a novel functional link between HA-CD44 interaction and BMP-induced Id1 and Id3 protein in melanoma. Targeting Id1 and Id3 protein expression by perturbation of the HA-CD44 interaction might be a promising and effective approach for melanoma therapy.

## Materials and Methods

### Cell culture and BMP stimulation

The murine melanoma cell line B16-F10 was cultured in DMEM (Thermo Fisher Scientific, Darmstadt, Germany) supplemented with 10% FCS (Thermo Fisher Scientific, Darmstadt, Germany) and 1% penicillin/streptomycin (Thermo Fisher Scientific, Darmstadt, Germany). The murine melanoma cell line Ret^46^ was grown in RPMI (Thermo Fisher Scientific, Darmstadt, Germany) containing 10% FCS and 1% penicillin/streptomycin. Both cell lines were cultivated at 37 °C in a 5% CO_2_ atmosphere. Stimulation of tumor cells with mouse recombinant BMP4 or BMP7 (R&D Systems, Wiesbaden-Nordenstadt, Germany) was performed with the indicated concentrations. Bovine testis hyaluronidase (BTH) and 4-MU sodium salt were purchased from Sigma-Aldrich, Taufkirchen, Germany. High molecular weight HA (Healon5) was from Abbott Medical Optics (Uppsala, Sweden).

### Cell proliferation assay

The CyQuant NF cell proliferation assay kit (Invitrogen, Karlsruhe, Germany) was used to assess the effect of mouse recombinant BMP4 or BMP7 or 4-MU on cell proliferation. B16-F10 or Ret cells were seeded at a density of 2000–4000 cells/well in a 96-well plate and treated with BMP4 or BMP7 (0–100 ng/ml) or 4-MU (0–5 mM) for 48 hours or 72 hours. Subsequently, the cells were incubated with 50 µl CyQuant Dye Binding Solution at 37 °C for 30 minutes. The fluorescence intensity of each well was measured with excitation at 480 nm and emission at 530 nm by using a fluorescence Infinite 200 PRO microplate reader (Tecan, Männedorf, Switzerland).

### HABP staining

Cells were plated on glass coverslips and incubated for 72 hours. BTH treatment was performed 90 min or 24 hours before analysis as indicated, then the medium was changed prior to BMP stimulation. Where indicated, cells were treated with the indicated concentrations of 4-MU 48 hours prior to analysis. For HABP staining, cells were fixed with 3.7% formaldehyde-PBS, 70% ethanol, and 5% glacial acetic acid (all v/v) for 15 min at room temperature and subsequently air-dried. Cells were washed three times with PBS. Blocking was performed using 3% BSA (biotin-free) in PBS for 1 hour at room temperature. Hyaluronic acid was stained with hyaluronic acid binding protein (HABP, Calbiochem #385911, 2.5 µg/ml) in 3% BSA overnight at 4 °C. Cells were washed three times with PBS. HABP was detected using Alexa Fluor 546-labelled Streptavidin (5 µg/ml) at room temperature for 1 hour. Cells were washed three times with PBS and stained with DAPI (0.5 µg/ml for 5 min). Slides were mounted with Fluoromount. Immunofluorescence images were acquired using an Axio Imager D1 microscope and a 63x objective (Zeiss, Germany). Total HABP staining signal (integrated density) was measured in at least 15 randomly selected fields per condition using ImageJ. The mean signal of fields stained with Alexa Fluor 546-labelled Streptavidin only (background) was subtracted, and the numbers were normalized to the mean value for the untreated cells, which was set to 1.

### RNA isolation and Reverse transcription PCR (RT-PCR)

Total RNA was isolated from the cells using TRIzol (Invitrogen, Karlsruhe, Germany) according to the manufacturer’s instructions. RNA (5 μg) was digested by 5 U DNase I (Thermo Fisher Scientific, Darmstadt, Germany) at 37 °C for 30 minutes. DNase I was inactivated by adding EDTA and heating at 65 °C for 10 minutes. After precipitation of the RNA with isopropanol, cDNA synthesis was performed using RevertAid H Minus reverse transcriptase and random primers (both from Thermo Fisher Scientific, Darmstadt, Germany). Negative controls containing no enzyme were set up for each sample. For standard semi-quantitative RT-PCR, cDNA templates were amplified with 30 cycles of denaturing at 95 °C for 15 sec, annealing for 30 sec and extension at 72 °C for 1 min. The following primers and annealing temperature were used: mouse HAS2, forward: 5′-ACAGGCACCTTACCAACAGGG-3′, reverse: 5′- GCATGCATAGATCAAAGTTCCCACG-3′ (66 °C); mouse HAS3, forward: 5′- ACTGCCTTCAAGGCCCTTGG-3′, reverse: 5′-AATGTTCCAGATGCGGCCAC-3′ (60 °C); mouse CD44, forward 5′-CCCACCATGGACCAAATGA-3′, reverse: 5′-GGTGCTCCGGATAAAGAAGGA-3′ (58 °C); mouse Rplp0, forward: 5′-GGACCCGAGAAGACCTCCTT-3′, reverse: 5′-GCACATCACTCAGAATTTCAATGG-3′ (60 °C). Amplicons were analysed using agarose gel electrophoresis using standard procedures.

### Quantitative RT-PCR (qRT-PCR)

Equal amounts of cDNA were mixed with SYBR Green PCR Master Mix (Thermo Fisher Scientific, Darmstadt, Germany) and 0.5 µM of each primer pair. qRT-PCR was conducted at 95 °C for 10 min, followed by 40 cycles of 95 °C for 30 s, 55 °C for 1 min, and 72 °C for 1 min using the Agilent Mx3005P QPCR System (Agilent, Santa Clara, CA, US). The specificity of the reaction was verified by analyzing melting curves. The sequences of primers used for qRT-PCR analysis of type I and type II BMP receptors are listed below. Rplp0 mRNA transcripts were served as an internal control. Relative quantitative analysis was performed using the ∆∆Ct method. ALK2, forward: 5′-GGCTGCTTTCAGGTTTATGAG-3′, reverse: 5′-TACTGCAAACACCACCGAGA-3′; ALK 3, forward: 5′-GGTCCTGCTGTCTCACTGGT-3′, reverse: 5′-GCTGTTCGGAGAAATTGGAA-3′; ALK6, forward: 5′-TCCAGAGCTTCGTAAGAGCA-3′, reverse 5′-ATTTGGCGCTGAGCTATGAC-3′; BMPR2, forward 5′-CTGCGGCTGCTTCGCAGAAT-3′, reverse: 5′-TGGTGTTGTGTCAGGAGGTGG-3′. ACVR2A, forward: 5′-TTGGTTCTGTCTCTTTCCCAA-3′, reverse: 5′-CGTTCGCCGTCTTTCTTATC-3′; ACVR2B, forward: 5′-GAAGATGAGGCCCACGATTA-3′, reverse: 5′-GGAGGTCACCAGAGAGACGA-3′.

### Western blot

Cells were lysed in radioimmunoprecipitation assay buffer (50 mM Tris, pH 7.4, 150 mM NaCl, 1 mM EDTA, 1% NP-40, 0.25% sodium deoxycholate) supplemented with EDTA-free Protease Inhibitor Cocktail (Roche, Mannheim, Germany). After sonication for 20 to 40 seconds at 50/60 Hz to break down chromosomal DNA, the lysates were centrifuged for 15 min at 13000 rpm. Total protein concentration was measured using the Pierce BCA protein assay kit (Thermo Fisher Scientific, Darmstadt, Germany). Proteins were diluted in sample buffer (final concentration: 2% SDS, 5% 2-mercaptoethanol, 10% glycerol, 0.002% bromophenol blue and 62.5 mM Tris-HCI pH 6.8, with 250 mM dithiothreitol added to the sample buffer freshly prior to use). The samples were subjected to SDS-PAGE and transferred to Immobilon-P PVDF Membrane (Merck Millipore, Darmstadt, Germany) by using standard Western blotting techniques. The membranes were probed with anti-human/mouse Id1 (clone 195–14, M086, 1 µg/ml) or Id3 (clone 17-3, M101, 1 µg/ml) rabbit monoclonal antibodies (CalBioreagents, California, USA), anti-ACTR II (F-12, sc-390977, 1 µg/ml, Santa Cruz Biotechnology, California, USA), and anti-CD44 (KM81, 1 µg/ml, hybridoma obtained from ATCC). The monoclonal anti-β-actin antibody (clone AC-15, #A5441, Sigma Aldrich, Taufkirchen, Germany) was used for a loading control. HRP-conjugated secondary antibodies were from DAKO (Hamburg, Germany). The protein bands were visualized using the Pierce ECL Western Blotting Substrate (Thermo Fisher Scientific, Darmstadt, Germany) for β-actin. AceGlow Chemiluminescence Substrate (Peqlab, Erlangen, Germany) was used to detect Id1 and Id3 proteins. Densitometry analysis was carried out using ImageJ. Numbers represent background-subtracted integrated densities of Id1, Id3, and CD44 divided by corresponding background-subtracted integrated densities of loading control (β-actin) bands. The numbers were normalized to lane 1 (control), which was set as 100%. In case of IP experiments, numbers represent background-subtracted integrated densities of CD44 and ACVR2B, normalized to lane 1. Three independent experiments were conducted and representative results are shown.

### siRNA-mediated CD44 knockdown

CD44 small interfering RNA (siCD44) and scrambled siRNA (siScr) were purchased from Thermo Fisher Scientific (ID: s63661), Darmstadt, Germany, and Origene (ID: siRNA-27), Herford, Germany, respectively. Transfections were performed with Lipofectamine 2000 (Invitrogen, Karlsruhe, Germany) according to the manufacturer’s instructions. Total RNA was isolated for detecting transcription of CD44 24 hours after transfection with CD44 siRNA, and flow cytometry together with western blot analysis were performed at this time point to analyse surface and total expression of CD44 protein, respectively. The transfection efficiency was monitored using an expression plasmid for Green Fluorescent Protein (GFP) through fluorescence microscopy and flow cytometry (Supplementary Fig. S5).

### Flow cytometry

Cells were detached with Ca^2+^/Mg^2+^ (-) DPBS and washed twice with Ca^2+^/Mg^2+^ (-) DPBS containing 10% FCS. The cells were resuspended with Ca^2+^/Mg^2+^ (-) DPBS containing 10% FCS. The cells were then incubated with the rat anti-mouse CD44 monoclonal antibody (KM81, 5 µg/ml, hybridoma obtained from ATCC) or isotype-matched control rat IgG (MAB006, 5 µg/ml, BD Biosciences, Heidelberg, Germany) for 1 hour at 4 °C, then were incubated with the secondary antibody for 30 minutes after three washes with PBS. Finally, FACS measurements were performed with the single cell suspension after washing a further three times with PBS. FACS analysis was performed using a FACSCanto II (BD Biosciences, Heidelberg, Germany) to determine GFP or CD44 expression. Dead cells were excluded using SytoxRed (Invitrogen, Karlsruhe, Germany). Unstained cells and cells incubated only with isotype control or secondary antibody were used as a negative control.

### Co-immunoprecipitation

B16-F10 and Ret cells were incubated with HA and/or BMPs as indicated. Cells were lysed in 30 mM Tris-HCl, 150 mM NaCl, 1 mM EDTA, 0.5% Triton-X-100, and 0.5% sodium deoxycholate. Cell lysates were placed on ice for 20 minutes, briefly sonicated, then centrifuged at 13000 g at 4 °C for 20 minutes. Pre-clearing was performed by incubating the cell lysates with Protein G-sepharose beads (Merckmillipore, Darmstadt, Germany), followed by centrifugation to remove the beads. The PureProteome Protein A/G Mix Magnetic Bead suspension (Merckmillipore, Darmstadt, Germany) was first incubated with an anti-ACVR2 polyclonal antibody (H-65, sc-25451, 0.5 µg/ml, Santa Cruz Biotechnology, CA, USA), with an anti-CD44 monoclonal antibody (clone IM7, #550538, 0.25 µg/ml, BD Biosciences, Heidelberg, Germany), or with an isotype-matched control IgG at room temperature for 30 minutes. The magnetic beads were then mixed with a 250 µl volume of 5 mM bis (sulfosuccinimdyl) suberate crosslinker solution at room temperature for 30–60 minutes. The pre-cleared cell lysates were then incubated with the magnetic beads at 4 °C overnight. Magnetic beads were separated from the lysate using a magnetic field, and washed four times with PBS. The magnetic beads were finally resuspended in 50 µl sample buffer and heated at 70 °C for 10 minutes. The supernatant was subjected to SDS-PAGE and Western blotting.

### Statistical analyses

Comparison between two groups in the proliferation assay and quantification of HABP stainings was performed using the Student’s t-test. Statistical significance was set at *p* < 0.05.

## Electronic supplementary material

Supplementary Information

## Data Availability

Materials, data and associated protocols will be made promptly available to interested readers without undue qualifications in material transfer agreements.
