# Author Correction: Hyaluronic acid-CD44 interactions promote BMP4/7-dependent Id1/3 expression in melanoma cells

**DOI:** 10.1038/s41598-019-55812-5

**Published:** 2019-12-11

**Authors:** Ruo-Lin Wu, Georg Sedlmeier, Lenka Kyjacova, Anja Schmaus, Julia Philipp, Wilko Thiele, Boyan K. Garvalov, Jonathan P. Sleeman

**Affiliations:** 1 European Center for Angioscience (ECAS), Medical Faculty of Mannheim, Heidelberg University, 68167 Mannheim, Germany; 2 KIT Campus Nord, Institute for Toxicology and Genetics, 76344 Karlsruhe, Germany; 3 Present Address: Department of Hepatopancreatobiliary Surgery and Organ Transplantation Center, First Affiliated Hospital of Anhui Medical University, Hefei, 230022 Anhui China

Correction to: *Scientific Reports* 10.1038/s41598-018-33337-7, published online 08 October 2018

In Figure 5 there are errors in the labelling of the western blots. The correct Figure 5 appears below as Figure 1.

**Figure 1:**
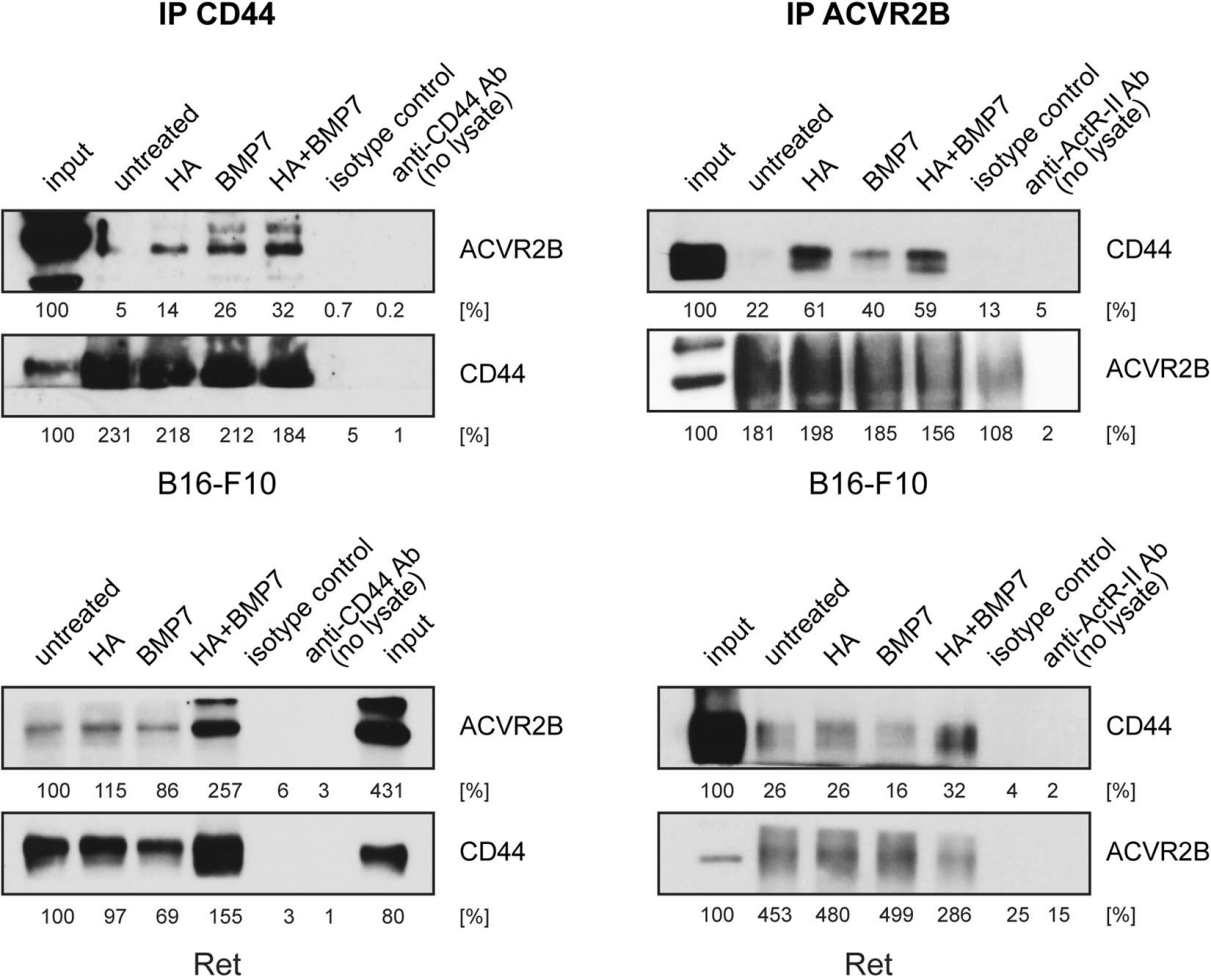
.

