# Supplementary material for: Hyaluronic acid-CD44 interactions promote BMP4/7-dependent Id1/3 expression in melanoma cells
Source: Sci Rep. 2018 Oct 8;8:14913. doi: 10.1038/s41598-018-33337-7 (PMC6175841; doi:10.1038/s41598-018-33337-7)
Supplement: Supplementary file 1 — Supplementary Information [file 41598_2018_33337_MOESM1_ESM.pdf]

Title page

Title: Hyaluronic acid-CD44 interactions promote BMP4/7-dependent Id1/3 expression in melanoma cells

Authors: Ruo-Lin Wu[1†], Georg Sedlmeier[1†], Kyjacova Lenka[1], Anja Schmaus[1, 2], Julia Philipp[1], Wilko Thiele[1, 2], Boyan Garvalov[1] and Jonathan P. Sleeman[1,2 *]

Authors' affiliations:

[1] European Center for Angioscience (ECAS), Medical Faculty of Mannheim, Heidelberg University, 68167 Mannheim, Germany

[2] KIT Campus Nord, Institute for Toxicology and Genetics, Karlsruhe, Germany

[†] Equal contribution

*To whom correspondence should be addressed:
Jonathan P. Sleeman,
University of Heidelberg, Medical Faculty Mannheim,
Centre for Biomedicine and Medical Technology Mannheim (CBTM)
TRIDOMUS-Gebäude Haus C
Ludolf-Krehl-Str. 13 – 17,
68167 Mannheim,
Germany.
Phone: +49 621 3839955
Fax: +49 621 383 9961.
E-mail: sleeman@medma.uni-heidelberg.de

Keywords: bone morphogenetic protein, inhibitor of differentiation protein, hyaluronic acid, CD44, melanoma cells

Total numbers of Figures: 13

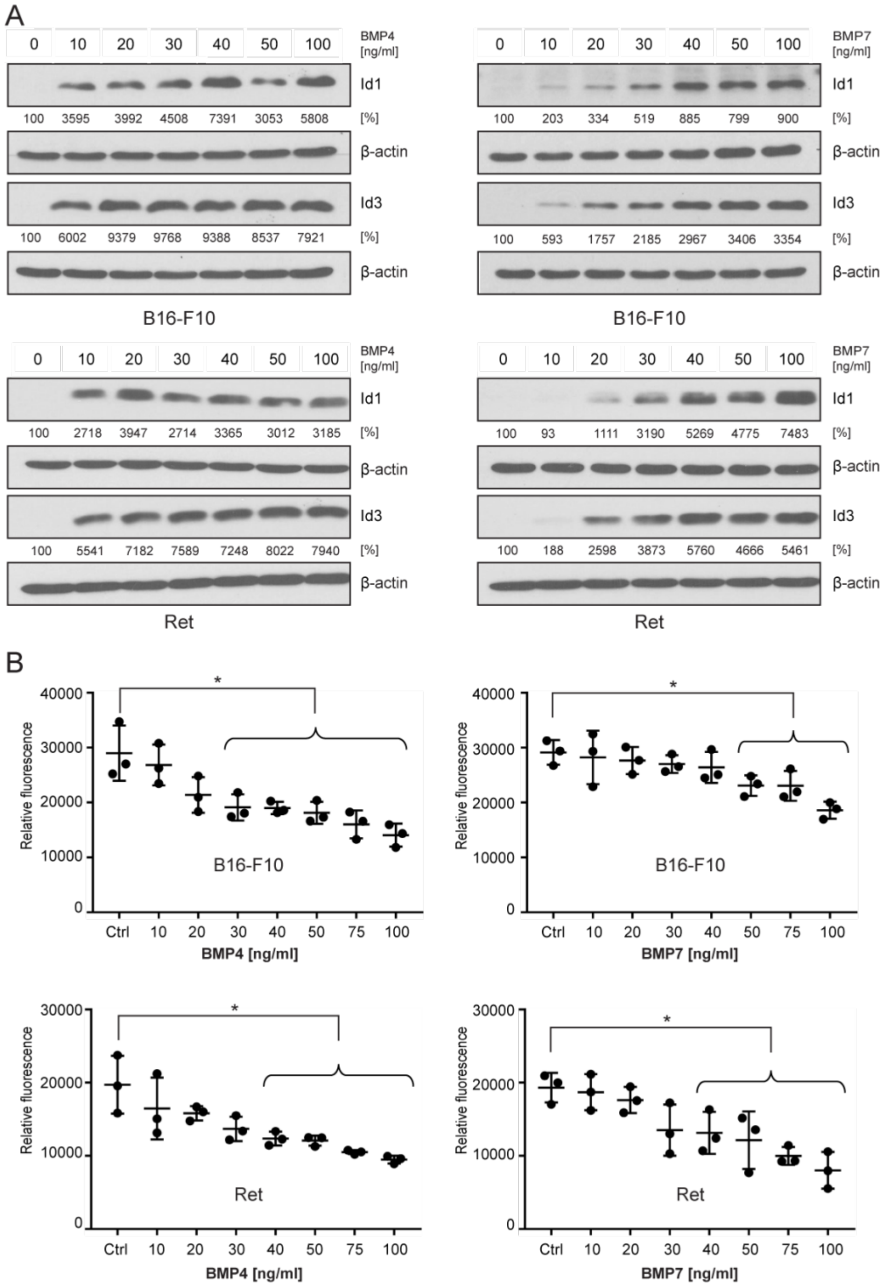

Supplementary Figure 1. BMP stimulation induces Id1/3 expression in melanoma cells, and high concentrations of BMPs show inhibitory effects on cell proliferation. (A) B16-F10 and Ret cells were incubated with the indicated concentrations (0-100 ng/ml) of recombinant BMP4/7 for 48 hours. Cell lysates were subjected to analysis of Id1/3 protein expression by Western blot. β-actin was used as a loading control. Densitometric quantification is shown below the Western blots, representing the signal normalized to the loading control (β-actin), relative to the untreated controls. (B) B16-F10 and Ret cells were incubated with the indicated concentrations (0-100 ng/ml) of recombinant BMP4/7 for 48 hours. Cell numbers were

quantified using the CyQUANT assay. The mean and standard error of triplicate samples are shown. Student’s t-test: *$p<0.05$.

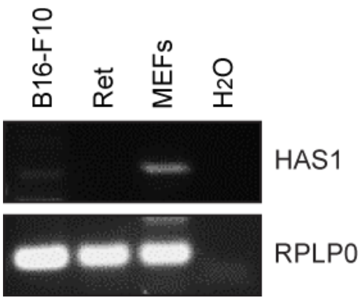

Supplementary Figure 2. Minimal HAS1 gene expression was found in B16-F10 cells, and no signal was detected in Ret cells by RT-PCR. Total RNA was isolated from B16-F10 and Ret cells, and RT-PCR was performed to analyze the gene expression of HAS1. cDNA isolated from mouse embryonic fibroblasts was used as a positive control. For a negative control, water instead of cDNA was added to the PCR reaction (H_2O). Rplp0 was used as an internal loading control.

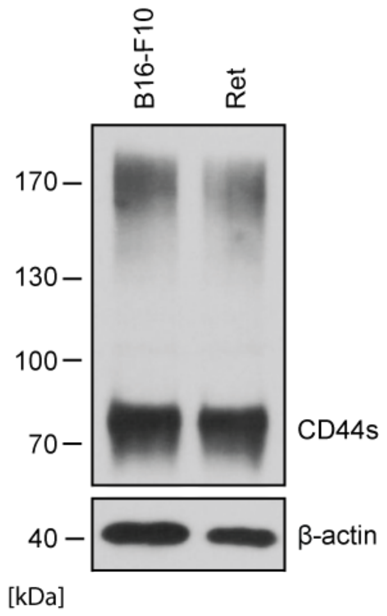

Supplementary Figure 3. Standard CD44 is predominantly expressed in B16-F10 and Ret melanoma cell lines. Cell lysates from B16-F10 and Ret cells were analyzed for CD44 standard (CD44s) and variant protein expression by probing Western blots with a pan-specific anti-CD44 antibody.

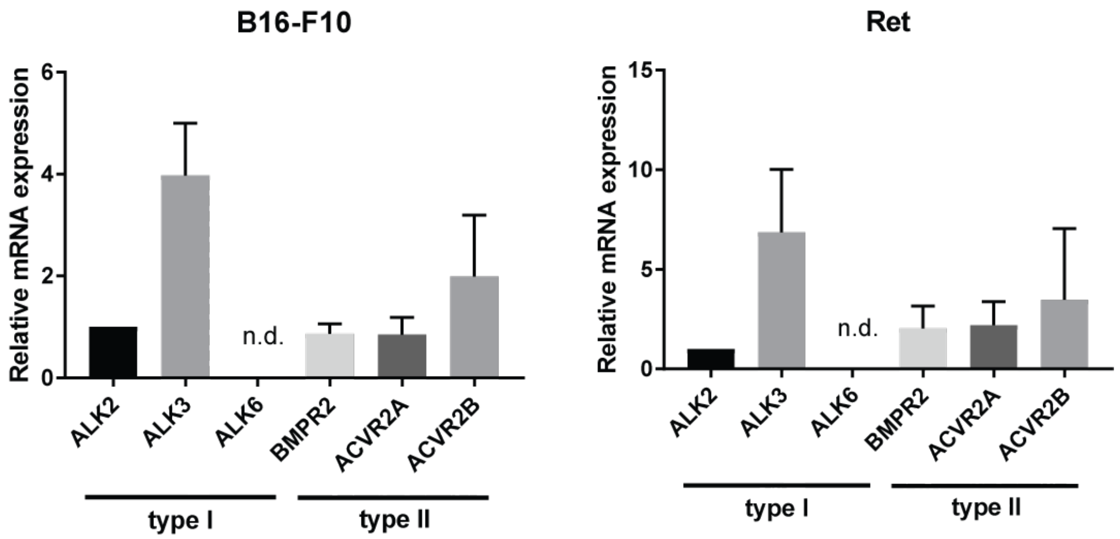

Supplementary Figure 4. ALK3 and ACVR2B are the main type I and type II BMP receptors, respectively, expressed on B16-F10 and Ret cells. Transcript levels of the three BMP type I (ALK2, ALK3, ALK6) and BMP type II (BMPR2, ACVR2A, ACVR2B) receptors were analyzed by qRT-PCR. ALK3 and ACVR2B exhibit the highest expression levels in both murine melanoma cell lines (B16-F10 and Ret). ALK6 mRNA levels were non-detectable (n.d.). The mean and standard deviation of three independent biological replicates is shown. Data are plotted relative to ALK2 mRNA transcript levels. Rplp0 was used as an internal loading control.

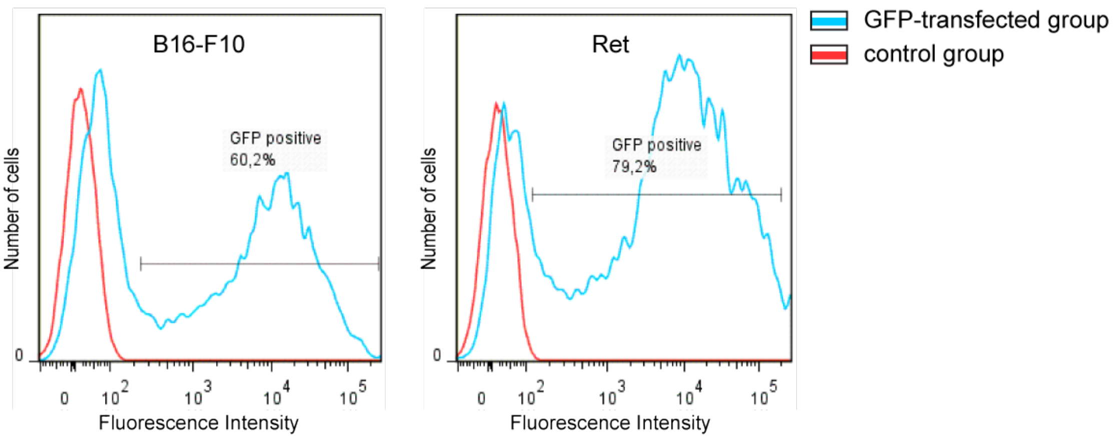

Supplementary Figure 5. Transfection efficiency was determined by FACS. B16-F10 and Ret cells were transiently transfected with or without a Green Fluorescent Protein (GFP) expression construct, then 24 hours after transfection the percentage of GFP-positive cells was quantified by FACS.

Fig. 1B

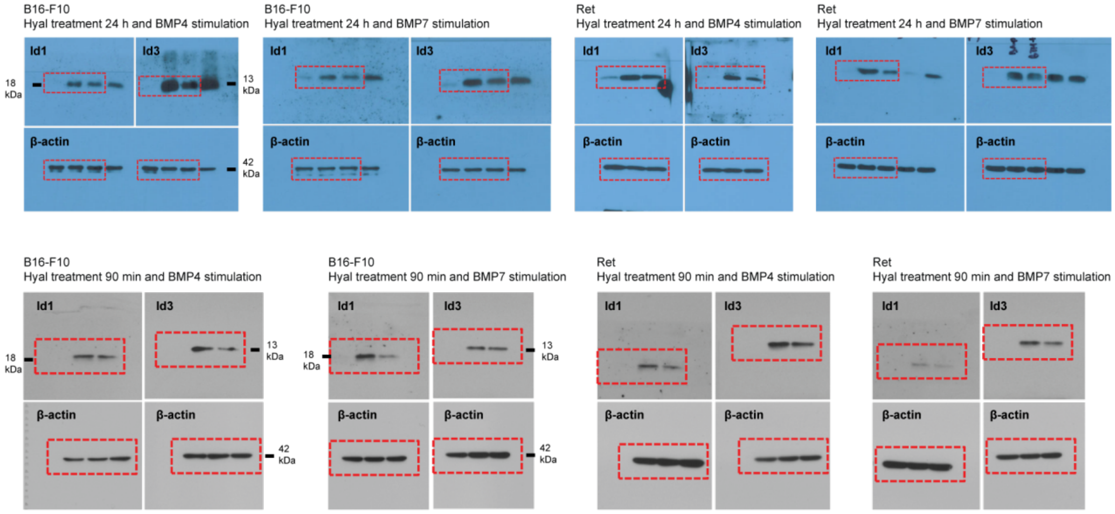

Fig. 2D

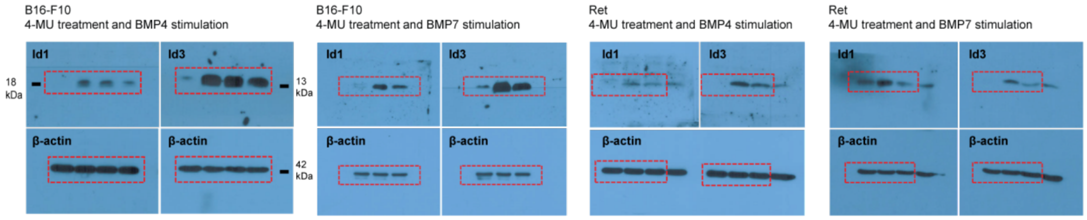

Fig. 3

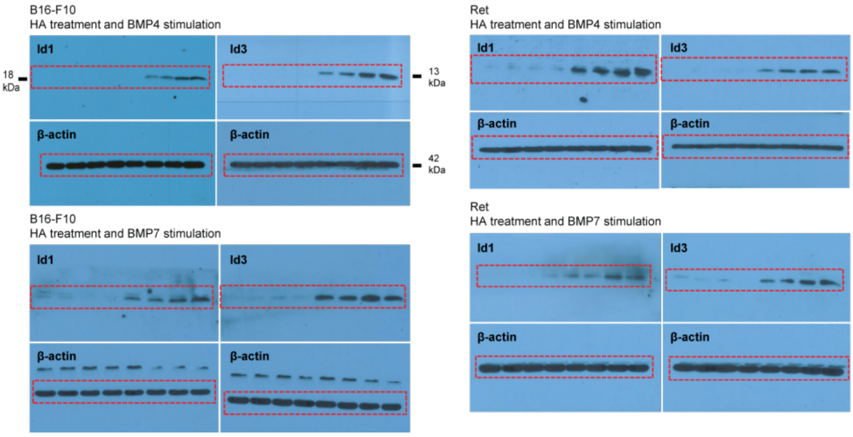

Supplementary Figure 6. Unprocessed scans of original western blots (part 1).

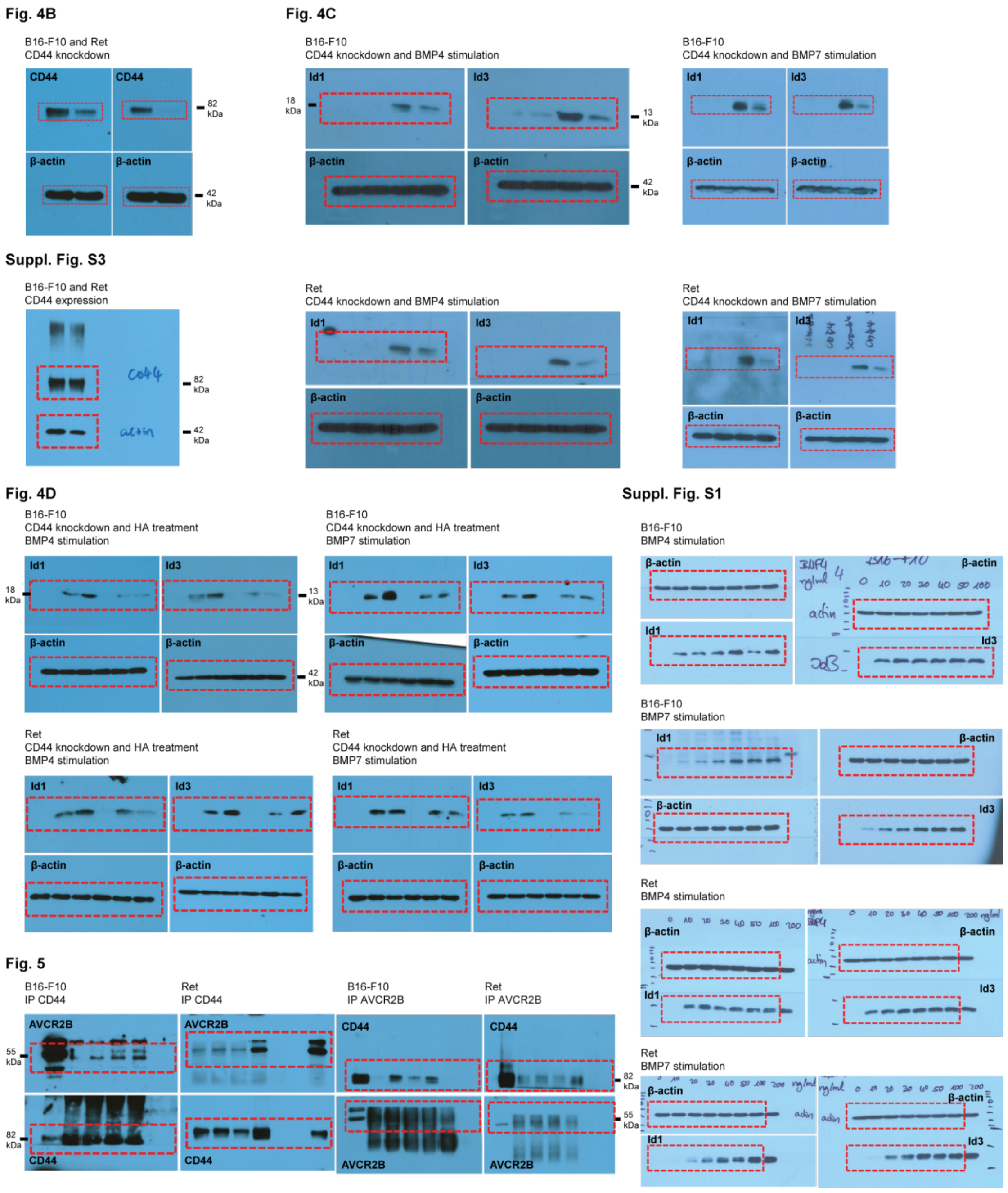

Supplementary Figure 7. Unprocessed scans of original western blots (part 2).
